# Hearing Impairment Among Drug-Resistant Tuberculosis Patients in Rural Eastern Cape: A Retrospective Analysis of Audiometric Findings

**DOI:** 10.3390/ijerph22050810

**Published:** 2025-05-21

**Authors:** Lindiwe Modest Faye, Mojisola Clara Hosu, Ntandazo Dlatu, Vatiswa Henge-Daweti, Teke Apalata

**Affiliations:** 1 Department of Laboratory Medicine and Pathology, Faculty of Medicine and Health Sciences, Walter Sisulu University, Mthatha 5117, South Africa; mhosu@wsu.ac.za (M.C.H.); vatiswa.henge-daweti@echealth.gov.za (V.H.-D.); tapalata@wsu.ac.za (T.A.); 2 Department of Public Health, Faculty of Health Sciences, Walter Sisulu University, Private Bag X1, Mthatha 5117, South Africa; ndlatu@wsu.ac.za

**Keywords:** audiometric assessment, adverse drug reaction, drug-resistant tuberculosis, hearing loss, ototoxicity, pure tone audiometry

## Abstract

Hearing loss (HL) is a major global health concern, with drug-induced ototoxicity contributing significantly, particularly in patients undergoing treatment for drug-resistant tuberculosis (DR-TB). In South Africa, where both TB and HIV are prevalent, the risk of treatment-related auditory damage is especially high. This study aimed to assess the prevalence and predictors of hearing impairment among DR-TB patients in rural Eastern Cape, South Africa. A retrospective analysis was conducted on 438 DR-TB patients treated between 2018 and 2020, using pure tone audiometry (PTA) to assess hearing status post-treatment. Demographic, clinical, and lifestyle data were extracted from patient records and analyzed using logistic regression. The overall prevalence of hearing loss was 37.2%. Risk was significantly associated with an older age, a male gender, DR-TB classification (MDR, pre-XDR, and XDR), unsuccessful treatment outcomes, and substance use. Prevalence of HL increased notably in patients aged 70 and older. Lifestyle factors, particularly combined use of tobacco, alcohol, and drugs, were linked to higher odds of HL. These findings underscore the need for routine audiometric screening and personalized treatment monitoring in DR-TB care, especially for high-risk populations. Early identification of ototoxicity risk factors can inform safer treatment regimens and improve patient outcomes in resource-limited settings.

## 1. Introduction

With an estimated yearly cost of over 750 billion dollars, hearing loss (HL) is the fourth leading cause of disability worldwide. It affects society and the economy and causes social isolation, loneliness, stigma, lower standard of living, and a reduction in personal productivity, endangering family prosperity and job stability [1,2]. HL has been associated with the treatment of drug-resistant tuberculosis (DR-TB), primarily due to the ototoxic effects of the medications used. Studies show that aminoglycosides (AGs) such as kanamycin and amikacin are among the most ototoxic drugs used in DR-TB regimens, causing irreversible damage to the inner ear’s sensory cells, particularly with prolonged exposure and higher doses [3]. This ototoxic effect is dose-dependent, with cumulative exposure increasing the risk of permanent hearing impairment [4]. Studies indicate that the prevalence of HL among patients receiving these treatments can be as high as 41% to 50% [5,6,7]. A study in Nigeria found that 89.5% of patients treated for DR-TB experienced hearing impairment after three months of treatment, with a significant increase from a baseline prevalence of 73.7% [8]. Additionally, second-line drugs, including linezolid, although less ototoxic, have also been associated with auditory side effects, indicating a need for comprehensive ototoxicity assessment in DR-TB patients. The mechanism of ototoxicity is linked to the damage these drugs inflict on the sensory hair cells in the cochlea, particularly affecting high-frequency hearing first, which can progress to lower frequencies over time [9,10].

Ototoxicity is the term used to describe the functional damage of the ear, particularly to the cochlea or auditory nerve and, occasionally, the vestibular system, brought on by drugs and chemicals [2,11]. It is one of the most reported adverse drug reactions (ADRs) in DR-TB management, with an increased risk in patients taking HIV treatment, thereby affecting the quality of life, treatment adherence, and, hence, treatment outcomes [12,13,14]. The cumulative dose of AGs has also been associated with an increased risk of HL, indicating that dose management is critical in preventing ototoxicity. Early detection through audiometry assessments and therapeutic drug monitoring (TDM) may prevent the risk of permanent damage [15]. Khosa-Shangase and Stirk [16] identified a gap concerning the audiometry assessments and management of ototoxicity in cases where it has been identified. The availability of evidence-based management protocols was lacking. A recent study by Stevenson et al. revealed similar findings of the need to reassess current guidelines and protocols to be more appropriate for community-based ototoxicity programs [17]. With one of the highest rates of multidrug-resistant TB (MDR-TB) in the world and prior use of AGs in TB management, South Africa is one of the countries where these detrimental effects of irreversible HL can have a severe impact on social and economic well-being [18]. Previous studies in South Africa have reported alarmingly high rates of pre-existing hearing loss in DR-TB patients even before initiating treatment. For example, studies by Davids and Crowley [18] documented a 60% baseline HL rate among patients in the Eastern Cape and KwaZulu-Natal, while a separate study from the Western Cape reported a 57% prevalence [11,19]. These findings emphasize the need for early audiometric screening and careful consideration of ototoxic drug regimens in this population. Following the reported cases of high prevalence of ototoxicity associated with AGs, the World Health Organization (WHO) recommended the replacement of AGs with bedaquiline (BDQ) and linezolid (Lzd) based regimens. These newer DR-TB drugs are known to be less associated with HL [19]. Given the risks associated with AG treatment, recent studies advocate regular audiometric assessments for DR-TB patients throughout their treatment course to detect early signs of HL and allow for timely intervention [15,19,20,21,22].

The primary aim of this study is to assess the prevalence and degree of hearing impairment among patients undergoing treatment for DR-TB and to identify key risk factors associated with HL in this population. Furthermore, the study investigated how demographic factors, like age and gender impact hearing outcomes, evaluate the influence of clinical variables such as DR-TB classification (e.g., multidrug-resistant (MDR) and extensively drug-resistant (XDR)), treatment outcomes, and comorbid conditions (e.g., HIV, hypertension, and diabetes), and assess the role of lifestyle factors, including smoking, alcohol use, and occupational noise exposure, in contributing to hearing impairment. Additionally, this study seeks to analyze the interactions among these factors to understand how combined risks may heighten susceptibility to HL during DR-TB treatment.

## 2. Materials and Methods

The research investigation evaluated auditory outcomes in individuals receiving treatment for DR-TB to determine the prevalence of auditory deficits, associated risk factors, and concurrent comorbidities from January 2018 to December 2020. This investigation was carried out across four TB clinics and a one referral hospital in the Oliver Reginald Tambo District Municipality, located in the Eastern Cape Province, South Africa. These selected clinics were identified as crucial primary care establishments for the diagnosis and treatment of TB, with a particular emphasis on hearing loss in DR-TB patients in rural and underprivileged communities experiencing a significant disease burden. The referral hospital was incorporated into the research due to its specialized function in the management of intricate DR-TB cases, which often involve severe comorbid conditions and necessitate the application of advanced diagnostic methodologies. The research methodology encompasses data gathering, variable selection, and statistical analysis aimed at thoroughly examining the risk factors for hearing impairment among patients with DR-TB, emphasizing the impact of age, treatment outcomes, comorbidities, and lifestyle determinants on auditory health.

### 2.1. Study Population and Data Collection

Patients diagnosed with DR-TB and treated at designated healthcare facilities over a specified study period were included. Inclusion criteria required that patients had completed both DR-TB treatment and audiometric assessments before, during, or after treatment. Patients with incomplete audiometric records were excluded. Patient demographic and clinical data were collected from electronic health records (EHRs), including age, gender, DR-TB classification (e.g., MDR and XDR), treatment outcome, comorbidities (e.g., HIV, hypertension, and diabetes), and lifestyle factors such as smoking and alcohol use.

### 2.2. Operational Definition

The following definitions were adapted from Faye et al. [23]:

Rifampicin-resistant tuberculosis (RR-TB): TB resistant to rifampicin but susceptible to isoniazid.

MDR-TB: The MDR-TB category in our analysis refers specifically to patients with tuberculosis that is resistant to both isoniazid and rifampicin but remains susceptible to fluoroquinolones and second-line injectable agents.

Pre-XDR-TB: A type of MDR-TB resistant to isoniazid, rifampin, and either a fluoroquinolone or a second-line injectable.

XDR-TB: A type of MDR-TB resistant to isoniazid, rifampin, fluoroquinolone, and either a second-line injectable or additional drugs like bedaquiline or linezolid.

Isoniazid resistant tuberculosis (INH-R-TB): TB resistant to isoniazid but susceptible to rifampicin.

### 2.3. Audiometric Testing

Audiometric assessments were conducted by a clinician using standardized hearing tests to measure hearing thresholds across a range of frequencies. Inclusion criteria required that patients complete audiometric assessments at either the baseline (pre-treatment) or after the completion of DR-TB treatment; mid-treatment assessments were not consistently available and were therefore excluded from analysis. The main objective of this assessment was to evaluate drug-induced hearing loss in patients with drug-resistant tuberculosis (DR-TB) using pure tone audiometry (PTA) as the standard diagnostic tool. Pure tone audiometry (PTA) was conducted across octave frequencies from 250 Hz to 8 kHz (250, 500, 1000, 2000, 4000, and 8000 Hz) using both air and bone conduction. Hearing impairment was defined as a hearing threshold greater than 25 dB HL at two or more contiguous frequencies in either ear. Based on this criterion, patients were categorized as having either “hearing impairment” or “normal hearing.” An air–bone gap of ≤10 dB across affected frequencies was used to classify hearing loss as sensorineural. Due to the study’s retrospective nature and the lack of consistent otoscopic or tympanometry data, definitive classification of conductive or mixed hearing loss was limited. Nonetheless, most audiometric profiles were consistent with bilateral high-frequency sensorineural hearing loss (SNHL), typically indicative of ototoxicity. The classification of “positive” versus “negative” audiometric results was based on post-treatment PTA assessments, which were consistently available for the entire study cohort. Patients with unilateral or bilateral hearing loss that met the threshold criteria were categorized as having a positive audiometric result. Baseline audiometry data were excluded from regression analyses due to incomplete documentation.

### 2.4. Variable Selection and Interaction Analysis

Key variables selected for analysis included age, gender, DR-TB type (e.g., RR-TB, MDR-TB, and pre-XDR), treatment outcome, HIV, and lifestyle factors (smoking and alcohol use). These were identified as potential risk factors influencing hearing outcomes. The study incorporated interaction terms in statistical models to explore the combined effect of variables on hearing outcomes. MDR-TB is defined as tuberculosis resistant to at least isoniazid and rifampicin. Pre-XDR-TB and XDR-TB are subcategories of MDR-TB. Pre-XDR-TB includes MDR-TB with additional resistance to either a fluoroquinolone or a second-line injectable. XDR-TB includes MDR-TB with additional resistance to both a fluoroquinolone and either a second-line injectable or newer drugs such as bedaquiline or linezolid.

### 2.5. Statistical Analysis

Frequencies, proportions, and means were calculated for demographic and clinical characteristics, with audiometry results summarized by age group, gender, DR-TB type, and comorbidity status. Lifestyle variables (smoking, alcohol, and drug use) were grouped into mutually exclusive categories. The reference group included patients who reported no use of any substances. The significance threshold was set at *p* < 0.05. Logistic regression was conducted to identify factors associated with hearing impairment. All statistical analyses were conducted using IBM SPSS Statistics for Windows, version 27.0 (IBM Corp., Armonk, NY, USA).

## 3. Results

**Figure 1:**
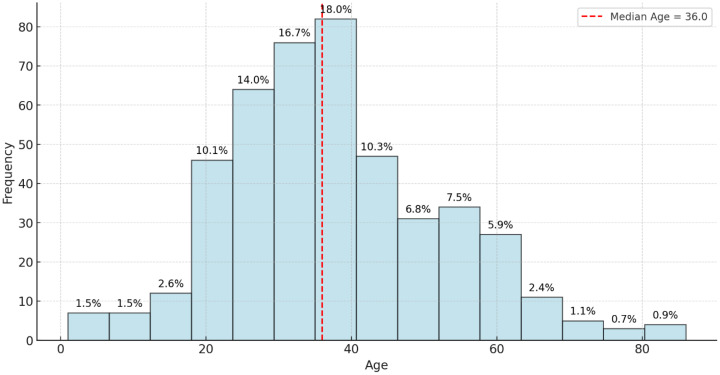
Age distribution of patients.

**Figure 2:**
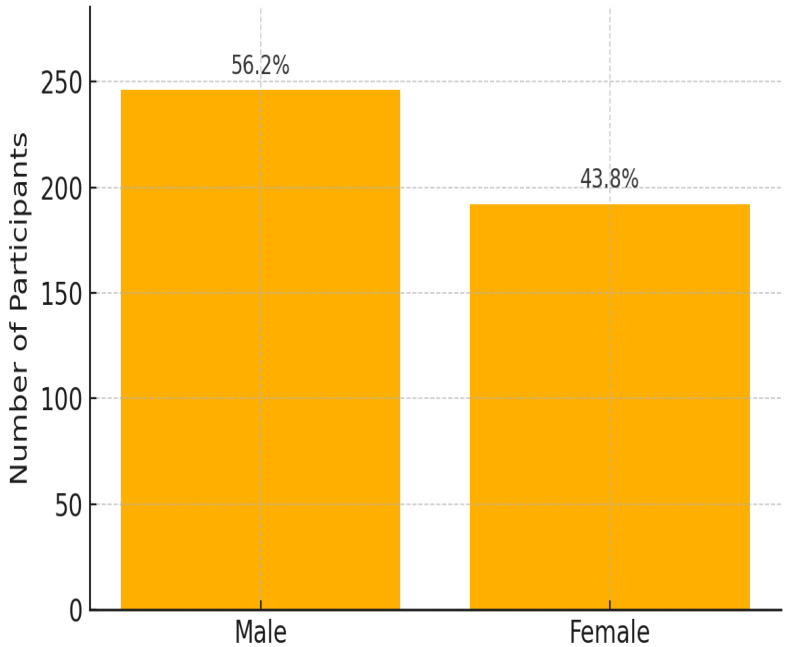
Gender distribution of DR-TB patients included in the final analysis (n = 438, excluding 18 patients with incomplete audiometric data).

This study reviewed records of 456 patients with DR-TB. Among these, 18 were excluded because of incomplete data on audiometry testing. Thus, 438 participants were selected and retained for the final analysis. Among the 163 patients with hearing impairment, 44.2% had mild HL, 28.2% had moderate HL, 16.0% had moderately severe HL, 9.2% had severe HL, and 2.5% had profound HL. Most cases involve bilateral high-frequency SNHL consistent with ototoxicity. Of the 438 participants whose records were reviewed, the prevalence of HL was 37.2%. The study participant’s mean age (±SD) was 37.8 (±14.8) years; the median age was 36 years, with ages ranging between 1 and 86 years (Figure 1). Almost half of the patients were aged between 30 and 49 years (48.2%). Of the 438 participants included in the final analysis, 56.1% were male and 43.9% were females (Figure 2).

Ages were grouped into age bands for analysis. Analyzing audiometric test results across different age bands revealed distinct trends in hearing health. In the 0–29 age group, out of 141 individuals tested, 53 (approximately 37.6%) showed positive results, while 88 (approximately 62.4%) were negative, indicating that most in this group do not face significant hearing issues. Similarly, the 30–49 age band, which included 211 tested individuals, had 79 positive results (about 37.4%) and 132 negative results (62.6%), demonstrating a comparable distribution where most do not exhibit audiometric concerns, but a notable minority does. This suggests that hearing issues become noticeable but remain consistent with the younger cohort, signaling the potential onset of hearing challenges. In the 50–69 age group, the trend shifts slightly; among 76 tested individuals, 25 (32.9%) had positive results, and 51 (67.1%) were negative. This shows that while the majority still do not have hearing problems, the proportion of individuals with concerns remains significant, pointing towards an age-related increase in hearing concerns. The most notable shift occurs in the 70+ age band, with only 10 individuals tested but yielding 6 positive results (60.0%) and 4 negative results (40.0%) (Table 1). This sharp rise underscores the correlation between advancing age and the prevalence of hearing issues, emphasizing the importance of proactive hearing health measures and frequent assessments in this older population. Overall, the results suggest relatively consistent hearing loss prevalence across age groups below 70, with a more pronounced increase observed only in the 70+ age category.

**Figure 3:**
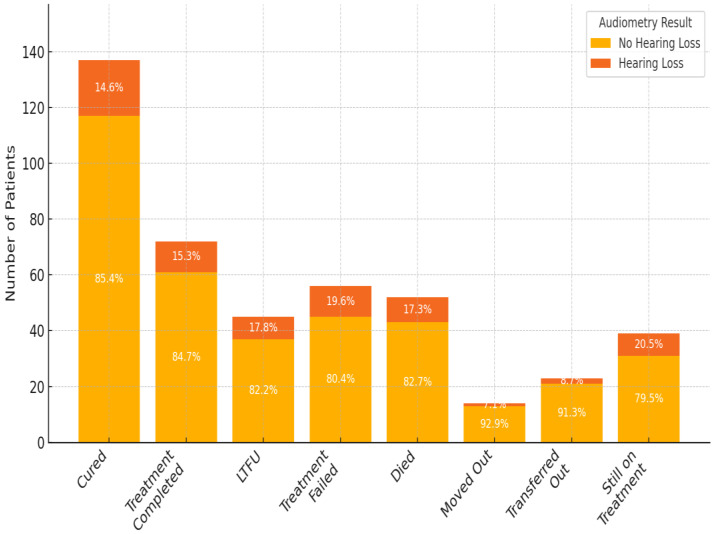
Distribution of audiometry results by treatment outcome among DR-TB patients (n = 438). Bar heights represent the number of patients per treatment outcome category. Each bar is divided by hearing status (positive = hearing loss, negative = no hearing loss). For the purpose of comparison in Figure 4, treatment outcomes were grouped as follows: ‘Cured’ and ‘Treatment Completed’ were classified as successful, while ‘Treatment Failed’, ‘Died’, ‘Lost to Follow-Up (LTFU)’, ‘Moved Out’, ‘Transferred Out’, and ‘Still on Treatment’ were grouped as unsuccessful outcomes. (Treatment outcomes: 1.0 = cured; 2.0 = treatment completed; 3.0 = LTFU; 4.0 = treatment failed; 5.0 = died; 6.0 = moved out; 7.0 = transferred out; and 8.0 = still on treatment).

Across the spectrum of treatment outcomes (n = 438), most patients exhibit negative audiometry results, suggesting a low prevalence of hearing impairment overall. Favorable outcomes, such as “Cured” and “Treatment Completed”, show particularly high proportions of negative results (85.4% and 84.7%, respectively), indicating that HL is generally uncommon among these groups (Figure 3). Positive audiometry findings, indicating hearing impairment, are present but represent a minority across most outcomes. For instance, only 14.6% of cases in the “Cured” group display positive results, pointing to a small subset of patients who experience hearing impairment post-treatment. There is notable variability in results based on treatment outcomes. Groups like “LTFU” (Lost to Follow-Up), “Moved Out”, and “Transferred Out” show lower frequencies of positive results, such as a 7% positive rate in “Moved Out”, which may reflect the clinical consequences of treatment interruption or patient mobility rather than missing audiometric data, as all included participants had complete audiometry records. More severe outcomes, including “Treatment Failed” and “Died”, also show positive results, though not as prominently, possibly because other health complications are more immediate in these cases. Patients “Still on Treatment” primarily exhibit negative results, with 78.4% showing no signs of hearing impairment. This outcome might indicate that ongoing treatment has not yet led to significant audiometric issues, although hearing outcomes for this group may evolve by the end of their treatment. This distribution of results highlights a general trend of minimal hearing impairment among patients with favorable treatment outcomes, while specific groups with incomplete treatment show variability that could be influenced by factors such as data collection limitations or other health priorities.

**Figure 4:**
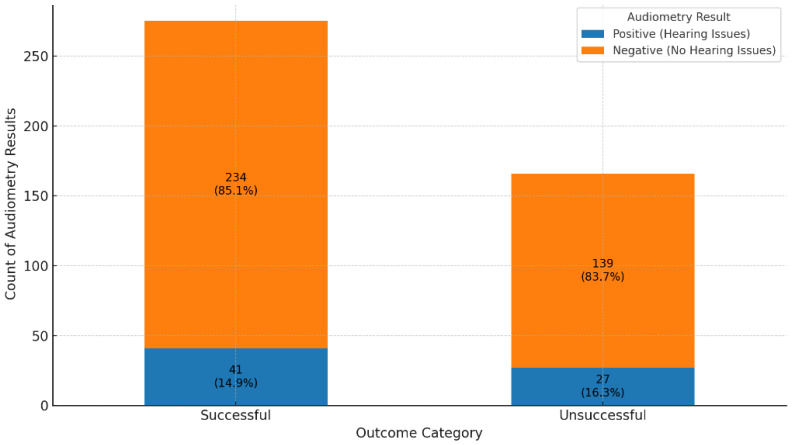
Comparison of audiometry results in successful versus unsuccessful treatment outcomes.

A comparison of audiometry assessments between patients with successful and unsuccessful treatment outcomes showed similar distributions of hearing loss (85.1% vs. 83.7% negative audiometry results, respectively), with no statistically significant differences (χ^2^ = 0.1459, *p* = 0.70). This finding suggests that hearing impairment was distributed similarly between patients with successful and unsuccessful treatment outcomes and does not indicate a significant association between treatment success and risk of audiometric issues.

The results of the chi-square test (chi-square statistic = 19.26, degrees of freedom = 7, *p*-value = 0.0074) indicate a statistically significant association between treatment outcomes and audiometry results. With a *p*-value of 0.0074, which is below the commonly accepted significance threshold of 0.05, we conclude that the distribution of hearing impairment (positive audiometry results) and no impairment (negative results) is not independent of treatment outcomes. This finding confirms that different treatment outcomes are meaningfully related to hearing status, suggesting that certain treatment results may be associated with either a higher or lower prevalence of hearing impairment.

**Figure 5:**
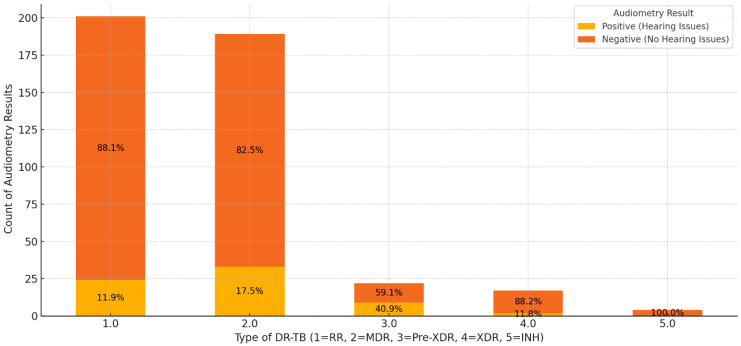
Distribution of audiometry results stratified by DR-TB type.

The combined results of audiometry assessments across different types of DR-TB highlight distinct trends in hearing outcomes. Among patients with RR-TB, 88.1% had negative audiometry results, suggesting that hearing issues are relatively rare in this group, with only 11.9% showing positive results (Figure 5). This indicates that while HL is present, it is not prevalent, though monitoring remains important. Patients with MDR-TB exhibited a higher proportion of positive results at 17.5%, with 82.5% showing no hearing issues, implying an increased risk of hearing impairment potentially linked to treatment intensity or disease impact. Although the prevalence of hearing loss appeared highest among pre-XDR patients (40.9%), followed by MDR (17.5%), XDR (11.8%), and RR-TB (11.9%), it is important to note that the pre-XDR, XDR, and INH-R-TB subgroups had relatively small sample sizes. As such, these percentages may be influenced by random variability and should be interpreted with caution. Notably, in INH-R-TB, all patients showed negative results, suggesting that this type poses minimal risk to hearing, possibly due to the nature of the resistance or fewer reported cases. The observed differences in hearing loss across DR-TB types suggest a possible trend, with higher rates among pre-XDR patients. However, given the small number of patients in the pre-XDR, XDR, and INH-R-TB categories, these differences should be interpreted cautiously. The apparent variations may reflect sampling variability rather than true underlying differences, and further research with larger samples is needed to confirm these patterns.

**Figure 6:**
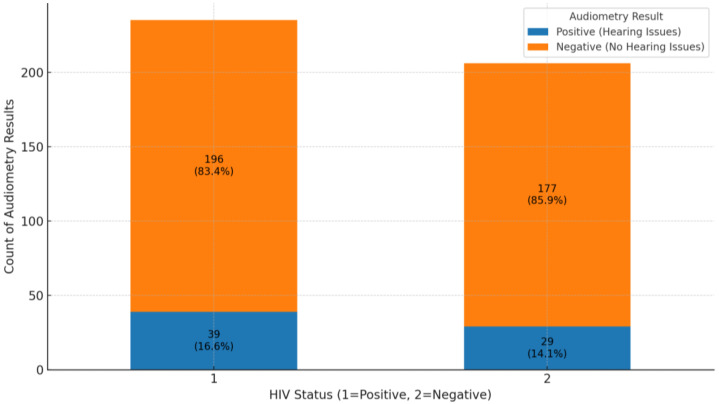
Comparison of audiometry results stratified by HIV status.

The analysis of audiometry results for HIV-positive and HIV-negative groups reveals important findings regarding hearing health. In the HIV-positive group, 196 out of 235 individuals (83.4%) had negative audiometry results, indicating no significant hearing issues, while 39 individuals (16.6%) had positive results, showing some level of hearing impairment (Figure 6). This suggests that while most HIV-positive individuals do not experience hearing problems, there is a notable portion that does. In the HIV-negative group, 177 out of 206 individuals (85.9%) also had negative results, with 29 individuals (14.1%) showing positive results. This indicates a similar trend where most HIV-negative individuals do not face hearing issues, although the percentage of HL is slightly lower than in the HIV-positive group. Overall, both groups show that most individuals do not suffer from hearing issues, as negative audiometry results dominate. The comparison of audiometry results between HIV-positive and HIV-negative groups revealed similar proportions of individuals without hearing loss (83.4% and 85.9%, respectively). While the proportion of hearing loss was slightly higher in the HIV-positive group, the difference was minimal and not statistically significant, indicating that HIV status may not be a major determinant of hearing impairment in this study population.

The analysis in Table 2, revealed multiple significant factors associated with hearing impairment. Age was a contributing factor, with each additional year increasing the odds of hearing impairment by 4.6% (*p* = 0.040), aligning with expected patterns of age-related auditory decline. In the logistic regression model, the rifampicin-resistant TB (RR-TB) group served as the reference category against which the other subtypes (MDR-TB, pre-XDR-TB, and XDR-TB) were compared. While five DR-TB subtypes are depicted in Figure 5, the INH-R-TB group was not included in the logistic regression analysis due to the small number of cases (n = 6) and absence of any reported hearing loss, which precluded meaningful statistical comparison.

Gender differences were evident, with males having more than twice the odds of hearing impairment compared to females (OR = 2.06, *p* = 0.010), suggesting potential biological, occupational, or lifestyle influences. In contrast, HIV status was not significantly associated with HL (*p* = 0.410), indicating that other clinical and lifestyle factors may play a more dominant role in audiometric outcomes.

Among patients with DR-TB, those with MDR-TB had 1.84 times higher odds of hearing impairment (*p* = 0.025), while those with pre-XDR and XDR-TB faced even greater risks (OR = 2.81, *p* = 0.003 and OR = 2.55, *p* = 0.009, respectively). This suggests a dose-dependent relationship, where more severe TB strains and their associated treatment regimens are linked to increased HL risk. Treatment outcome was another critical factor, with unsuccessful treatment significantly raising the likelihood of hearing impairment (OR = 3.08, *p* = 0.001). This finding suggests that prolonged or ineffective treatment regimens may exacerbate ototoxic effects or disease progression, increasing the risk of hearing damage.

Compared to patients who did not engage in any substance use, those who smoked and drank alcohol had 2.3 times higher odds of hearing loss (OR = 2.30, *p* = 0.016), while those who used all three (smoking, alcohol, and drugs) had more than triple the odds (OR = 3.39, *p* = 0.002).

## 4. Discussion

This study investigated HL prevalence and risk factors in patients undergoing DR-TB treatment, offering insights into age, treatment outcomes, DR-TB type, and comorbid conditions associated with audiometric outcomes in rural Eastern Cape, South Africa.

It is important to note that the prevalence and predictors of hearing loss reported in this study reflect post-treatment audiometric outcomes. Baseline assessments, while useful in identifying pre-existing HL, were excluded from multivariate analysis due to inconsistent documentation. In the clinical settings where this study was conducted, audiometric assessments were routinely reported using positive audiometry results to indicate hearing impairment and negative audiometry results to indicate the absence of hearing impairment. The study primarily relied on PTA, the standard diagnostic tool in these settings, to assess hearing thresholds at baseline and post-treatment. While objective assessments such as Distortion Product Otoacoustic Emissions (DPOAE) and Auditory Brainstem Response (ABR) could have provided additional insights into early cochlear damage, they were not included due to resource constraints. The findings revealed a considerable prevalence of hearing impairment among DR-TB patients, particularly in those with prolonged exposure to ototoxic drugs, highlighting the need for routine audiometric monitoring. Baseline audiometric evaluations are frequently not carried out in resource-constrained nations like South Africa before ototoxic damage is expected to develop [17,24], which results in an underdiagnosis of pre-existing HL [15]. Testing conducted at baseline to evaluate pre-existing comorbidities before treatment initiation showed 5.5% of participants with pre-existing HL [2]. Aminoglycoside (AG)-induced HL was detected in about a third (37.2%) of our participants. The findings of this study is evidenced by other reports suggesting the prevalence of pre-existing HL in the DR-TB patient cohort in South Africa. The study by Hong et al. conducted in the Eastern Cape and KwaZulu-Natal reported a 60% pre-existing HL before treatment, while another study conducted in the Western Cape of South Africa detected HL in 57% of patients [15,25]. Patients presenting with pre-existing HL before starting DR-TB treatment are particularly vulnerable to further HL after starting an AG regimen. Hence, the prevalence of pre-existing HL is a crucial factor for consideration in the South African Ototoxicity Monitoring Plans (OMPs) [17]. Previous studies have indicated that patients presenting with pre-existing hearing loss prior to DR-TB treatment initiation are particularly vulnerable to further deterioration due to aminoglycoside-induced ototoxicity [15,25]. Although our study did not analyze baseline HL status, this remains a critical consideration in ototoxicity monitoring and treatment planning. As reported in previous studies, patients with pre-existing hearing loss prior to DR-TB treatment initiation may experience a substantially increased risk—up to 8-fold—of further hearing deterioration during treatment [15,25]. In our study, audiometric results were assessed post-treatment as a binary outcome and therefore changes over time could not be evaluated. The increased risk of AG-induced HL in DR-TB patients with pre-existing HL is confirmed by the results of the current study, which indicated that patients presenting with a pre-existing HL at the time of the baseline assessment had an increase in hearing deterioration up to eight times higher than those with no pre-existing HL. There is a clear age-dependent increase in HL prevalence. Younger age groups (0–49) have predominantly negative audiometric results, indicating good hearing health, while those over 50, particularly the 70+ age group, show a dramatic rise in positive audiometry results, with HL affecting over half of this demographic. This pattern reflects age-related hearing deterioration, underscoring the need for proactive monitoring and early intervention in older DR-TB patients. In this study, age is a significant factor contributing to hearing impairment, with each additional year increasing the odds of experiencing such impairment by approximately 4.6% (*p* = 0.040). This aligns with established patterns of age-related auditory decline, which have been extensively documented in the literature. For instance, Haile et al. emphasized that nearly everyone will experience some degree of HL if they live long enough, with at least 50% likely to require intervention for moderate-to-complete HL as they age [25]. This trend is further supported by studies indicating that the prevalence of HL escalates with age, leading to a substantial increase in the number of individuals affected as the population ages [25,26]. The CONSTANCE study, a large cohort study conducted in France involving over 186,000 participants, found that the prevalence of HL significantly increases with age. While age-related hearing loss (ARHL) is expected in older adults, the prevalence of HL in our DR-TB population exceeds the global and regional averages for the same age groups. For example, the Global Burden of Disease (GBD) 2019 data indicate that HL affects approximately 20–25% of individuals aged 50–69 and around 45–50% of those aged 70 and above [25]. In contrast, our study found HL prevalence rates of 32.9% and 60.0% in these age groups, respectively. This suggests an additive or amplifying effect of ototoxicity associated with DR-TB treatment, especially in older adults who may already be vulnerable to ARHL. These elevated rates underscore the need for routine audiometric monitoring during treatment, particularly for older patients.

HL rates rose from 3.4% in individuals aged 18–25 to 73.3% in those aged 71–75 [27]. Consistent with our findings on the prevalence of HL in DR-TB patients over 50 years old, other studies support the observation that older age groups, particularly those over 50, experience a dramatic rise in hearing impairment. Age-related HL (ARHL), or presbycusis, is prevalent among older adults, with nearly one in three people between ages 65 and 74 experiencing HL, and this figure increases to nearly half for those over 75, particularly affecting over half of individuals in their 70s. ARHL is the most common sensory deficit in the elderly and typically presents as a progressive bilateral HL primarily affecting higher frequencies [27,28]. However, some studies emphasize that variability due to genetic and socioeconomic factors such as education level and access to healthcare may mitigate or exacerbate age-related declines in auditory health, suggesting that not all older adults will experience severe hearing impairments [29,30]. Understanding these nuances is critical for developing targeted interventions for different age groups within DR-TB populations to enhance their overall treatment outcomes and quality of life. Biological factors significantly contribute to the gender differences observed in hearing impairment. Research suggests that males may be more prone to certain types of hearing loss due to genetic and physiological variations. For example, hormonal influences and differences in ear anatomy can affect auditory processing and increase susceptibility to damage from ototoxic medications commonly used in tuberculosis treatment [31,32]. Furthermore, males may have a higher prevalence of age-related hearing loss, which could worsen the effects of tuberculosis-related hearing impairment. The study links treatment success with better hearing outcomes. Patients who achieved “Cured” or “Treatment completed” status had higher rates of negative audiometry results, suggesting minimal hearing impairment post-treatment. In contrast, groups with unsuccessful outcomes (e.g., “Lost to Follow-Up”, “Treatment Failed”, and “Died”) showed a higher incidence of HL, possibly due to prolonged illness, insufficient treatment, or disease complications. A study conducted on patients with ENT TB found that those who achieved a cured or treatment-completed status had significantly better hearing outcomes. Among 200 patients, those who were cured showed higher rates of normal audiometry results compared to those who experienced treatment failure. The study emphasized the importance of regular audiological assessments for patients undergoing anti-TB treatment, particularly those on ototoxic medications like AGs. This supports the notion that successful treatment correlates with better auditory health post-treatment [20]. Although the chi-square analysis across all eight treatment outcome categories revealed a statistically significant association with hearing loss (*p* = 0.0074), the simplified comparison between successful and unsuccessful outcomes did not reach significance (*p* = 0.70). This discrepancy may be explained by the loss of nuance when collapsing diverse outcome types into binary categories, potentially obscuring subgroup-specific risks. These mixed findings underscore the need for more detailed, outcome-specific analysis in future studies. A different study, however, found that regardless of the outcome of treatment, a considerable percentage of MDR-TB patients receiving large doses of AGs acquired HL. The ototoxic nature of the medications used in their regimen put even patients who had effective treatment outcomes at risk for auditory damage, as 70% of patients had some form of hearing impairment [15,30]. These results indicate that patients who achieve clinical success may experience substantial HL, thus challenging the belief that successful treatment is always associated with better hearing outcomes. This underscores the need for balance with careful monitoring and management strategies to mitigate auditory risks while ensuring effective TB treatment. While most HIV-positive and HIV-negative individuals maintained normal hearing, the HIV-positive group showed a slightly higher rate of HL. This difference suggests a marginal increase in risk associated with HIV, emphasizing the importance of regular hearing assessments in HIV-positive DR-TB patients. According to the prospective case-control study from Cameroon on the effect of HIV infection and highly active antiretroviral therapy on hearing function, HL is more frequent in HIV-infected patients compared with uninfected patients. The HIV-positive patients presented with otologic symptoms such as HL, dizziness, tinnitus, and otalgia compared with HIV-negative patients, where the difference was statistically significant. A total of 27.2% of patients with HL in the HIV-positive group, as compared to 5.6% in the HIV-negative group were reported. Compared with HIV-negative individuals, the odds of hearing loss were higher among HIV-infected patients who were either HAART-naive or receiving first- or second-line Highly Active Antiretroviral Therapy (HAART). Antiretroviral Therapy (ART) may interact with ototoxic TB medications, contributing to increased auditory risk [27]. However, in the current study, we did not report on the uptake of ART by HIV-positive participants. Social history factors show a strong connection to hearing impairment. Numerous studies align with our findings, indicating that hearing impairment is significantly influenced by various social determinants, particularly socioeconomic status (SES), social relationships, and health outcomes [33,34,35]. Research shows that lower SES is associated with higher rates of hearing impairment. For instance, individuals in the lowest income quintile have an odds ratio (OR) of 2.10 for hearing impairment compared to those in the highest income quintile [35]. Furthermore, studies suggest that lower education levels and a lack of health insurance contribute to an increased prevalence of hearing loss [34,35]. When compared to general population estimates, the prevalence of hearing loss among DR-TB patients in this study was substantially higher across all age groups. For instance, while global estimates from the GBD Study 2019 indicate hearing loss prevalence rates of 5–10% in adults aged 30–49, and up to 50% in those aged 70 and above, our findings showed much higher rates: 37.4% in the 30–49 age group and 60.0% in those aged 70+. This reinforces the compounded ototoxic risk associated with DR-TB treatment, particularly among older patients already predisposed to age-related hearing decline [28].

## 5. Conclusions

This study highlights the multifactorial nature of hearing loss in DR-TB patients, with several demographic, clinical, and lifestyle factors independently associated with increased risk. While successful treatment is linked to better hearing outcomes, patients with unsuccessful or incomplete treatments, advanced DR-TB, and specific comorbidities require closer audiometric monitoring. The findings underscore the need for targeted interventions and comprehensive care to reduce hearing impairment risks, particularly for high-risk groups. By identifying patients most vulnerable to HL, this study enables healthcare providers to implement early detection, personalized monitoring, and preventive strategies, ultimately preserving auditory health and improving quality of life. The insights gained can guide clinicians in optimizing treatment plans by considering ototoxic risks associated with demographic, clinical, and lifestyle factors. Additionally, as DR-TB remains a global health concern, these findings support the need for regular audiometric testing and the prioritization of less ototoxic treatment alternatives. Implementing alternative treatment options, such as newer, less ototoxic drugs like BDQ and delamanid, may also help mitigate the risk of HL while maintaining treatment efficacy.

## 6. Study Limitations

This study has several limitations that should be taken into account when interpreting the findings. Due to its retrospective design, the study relied on existing patient records, which resulted in limitations regarding data completeness and consistency, especially concerning audiometric assessments and otoscopic examinations. Furthermore, objective hearing assessments such as Distortion Product Otoacoustic Emissions (DPOAE) and Auditory Brainstem Response (ABR) were not included, which restricts our understanding of cochlear function and early-stage hearing loss. The findings are also specific to patients with Drug-Resistant Tuberculosis (DR-TB) treated at selected healthcare facilities, which may impact their generalizability to wider populations or different healthcare contexts. Additionally, although the study utilized the positive/negative audiometry reporting system that is standard in clinical practice, this approach may not fully comply with international audiological classification standards. Moreover, the study did not account for potential confounding factors such as genetic predisposition, long-term exposure to occupational noise, and other underlying medical conditions that may contribute to hearing loss. Lastly, while the study primarily focused on audiometric assessments conducted before and after treatment, it lacked long-term follow-up to evaluate the persistence or progression of hearing loss following the completion of treatment.

## 7. Recommendations

To enhance the detection, prevention, and management of hearing loss (HL) in patients with drug-resistant tuberculosis (DR-TB), we recommend the implementation of baseline and regular audiometric assessments throughout the treatment process. This approach will facilitate the early detection of ototoxicity and allow for timely interventions. Additionally, incorporating objective hearing tests such as Distortion Product Otoacoustic Emissions (DPOAE) and Auditory Brainstem Response (ABR) alongside Pure Tone Audiometry (PTA) in clinical practice will provide a more comprehensive evaluation of cochlear function and early-stage hearing loss. While continuing to use the existing positive/negative audiometry reporting system in clinical settings, efforts should also be made to integrate internationally recognized hearing loss classifications to enhance scientific interpretation and standardization.

## Figures and Tables

**Table 1:** Prevalence of audiometry results by age group.

Age Group	Positive Audiometry Result n (%)	Negative Audiometry Result n (%)
0–29	53 (37.6)	88 (62.4)
30–49	79 (37.4)	132 (62.6)
50–69	25 (32.9)	51 (67.1)
70+	6 (60)	4 (40)

**Table 2:** Logistic regression results for predicting hearing impairment.

Predictor	β (Log Odds)	Wald χ^2^	df	*p*-Value	Odds Ratio (95% CI)
Age (years)	0.045	4.21	1	0.040 *	1.046 (1.002–1.091)
Male gender	0.723	6.57	1	0.010 *	2.06 (1.19–3.56)
HIV positive	0.118	0.67	1	0.410	1.13 (0.73–1.78)
MDR-TB	0.612	5.03	1	0.025 *	1.84 (1.08–3.12)
Pre-XDR TB	1.032	8.91	1	0.003 **	2.81 (1.42–5.55)
XDR-TB	0.934	6.87	1	0.009 **	2.55 (1.26–4.98)
Treatment outcome (Unsuccessful)	1.124	10.31	1	0.001 **	3.08 (1.65–5.74)
Smoking	0.507	2.99	1	0.084	1.66 (0.94–2.94)
Alcohol use	0.219	1.21	1	0.270	1.25 (0.82–1.91)
Smoking and Alcohol use	0.831	5.79	1	0.016 *	2.30 (1.17–4.50)
Smoking, Alcohol, and Drug Use	1.221	9.62	1	0.002 **	3.39 (1.53–7.52)

* indicates *p*-value < 0.01 (not highly significant), ** indicates *p*-value < 0.01 (highly significant).

## Data Availability

Data can be requested from the corresponding author.

## Abbreviations

The following abbreviations are used in this manuscript:

TB	Tuberculosis
HL	Hearing loss
DR-TB	Drug-resistant Tuberculosis
MDR-TB	Multidrug-resistant tuberculosis
XDR-TB	Extensively drug-resistant tuberculosis
RR-TB	Rifampicin-resistant tuberculosis
INH-R-TB	Isoniazid-resistant tuberculosis
ADR	Adverse drug reaction
AGs	Aminoglycosides
BDQ	Bedaquiline
LZD	Linezolid
EHR	Electronic Health Records
PTA	Pure tone audiometry
LTFU	Loss to follow-up
DPOAE	Distortion Product Otoacoustic Emissions
ABR	Auditory Brainstem Response
OMP	Ototoxicity Monitoring Plan
ARHL	Age-related hearing loss
